# The Do's and Don'ts of Neurofeedback Training: A Review of the Controlled Studies Using Healthy Adults

**DOI:** 10.3389/fnhum.2016.00301

**Published:** 2016-06-17

**Authors:** Jacek Rogala, Katarzyna Jurewicz, Katarzyna Paluch, Ewa Kublik, Ryszard Cetnarski, Andrzej Wróbel

**Affiliations:** Laboratory of Visual System, Nencki Institute of Experimental Biology, Polish Academy of SciencesWarsaw, Poland

**Keywords:** neurofeedback training, EEG, replicability, protocol efficacy, methodology

## Abstract

The goal of EEG neurofeedback (EEG-NFB) training is to induce changes in the power of targeted EEG bands to produce beneficial changes in cognitive or motor function. The effectiveness of different EEG-NFB protocols can be measured using two dependent variables: (1) changes in EEG activity and (2) behavioral changes of a targeted function (for therapeutic applications the desired changes should be long-lasting). To firmly establish a causal link between these variables and the selected protocol, similar changes should not be observed when appropriate control paradigms are used. The main objective of this review is to evaluate the evidence, reported in the scientific literature, which supports the validity of various EEG-NFB protocols. Our primary concern is to highlight the role that uncontrolled nonspecific factors can play in the results generated from EEG-NFB studies. Nonspecific factors are often ignored in EEG-NFB designs or the data are not presented, which means conclusions should be interpreted cautiously. As an outcome of this review we present a do's and don'ts list, which can be used to develop future EEG-NFB methodologies, based on the small set of experiments in which the proper control groups have excluded non-EEG-NFB related effects. We found two features which positively correlated with the expected changes in power of the trained EEG band(s): (1) protocols which focused on training a smaller number of frequency bands and (2) a bigger number of electrodes used for neurofeedback training. However, we did not find evidence in support of the positive relationship between power changes of a trained frequency band(s) and specific behavioral effects.

## Introduction

Electroencephalogram (EEG) based neurofeedback (NFB) is a method in which brain activity is modulated via self-induced increases or decreases in the power of selected EEG frequency bands. The subject's control over his or her EEG activity is typically mediated with visual or auditory feedback. EEG-NFB is widely used as a therapy for certain mental, cognitive, and behavioral disorders (e.g. ADHD, for review see Arns et al., 2009); or as supportive training to improve cognitive performance (e.g. attention or memory, for review see Gruzelier, 2013a). Since the pioneering work of Sterman and Friar (1972), the number of publications devoted to EEG-NFB has systematically increased. During the first two decades (1972-1990), 162 NFB-based studies were published, based on a search in Google Scholar for the keyword ‘neurofeedback’. This number increased rapidly in the subsequent decades, reaching 1,260 in nineties and 6,100 between 2001 and 2010. Since 2011, there have been over 9000 publications devoted to various aspects of EEG-NFB. However, despite multiple promising case reports, reliable experimental research is scarce, and the methodologies and results inconsistent.

The validity of EEG-NFB protocols can be measured by unambiguous changes in EEG activity and by changes in the targeted cognitive function. Unfortunately, most of the work conducted in the EEG-NFB field has failed to satisfy the unambiguity criterion for both of the variables and the field itself has shown a big tolerance for violations of scientific methodology. Several reviews have been published which focused mainly on the clinical aspects of EEG-NFB training and summarized the protocols and their effects. One of the first reviews, published by Vernon et al. (2004), concerned the application of EEG-NFB for the treatment of attention deficit hyperactivity disorder (ADHD). The authors discussed various experimental variables (factors), namely, protocol, duration of the training, location of electrodes, signal modality and discussed their possible impact on treatment. The methodological findings of this review recommended visual rather than auditory feedback, with the best results obtained with visual and auditory modalities combined. In addition, the review suggested that at least 20 EEG-NFB sessions were necessary to achieve therapeutic effects, and that beta band/SMR protocols may play a role in successful ADHD treatment. However, studies examining EEG-NFB in the treatment of ADHD have raised important concerns about the validity of the treatment paradigm(s) mainly due to the lack of control groups or evidence of training specificity related to EEG changes (Vollebregt et al., 2014; Zuberer et al., 2015).

The first quantitative review of EEG-NFB methodology was reported by Arns et al. (2009) and focused on controlled ADHD studies. This review supported the view that positive therapeutic effects of EEG-NFB training could be achieved for all symptoms of ADHD. However, in contrast to Vernon's conclusions (Vernon et al., 2004), Arns concluded that inattention and hyperactivity were most sensitive to the non-specific treatment factors (e.g., therapist-patient interactions) and not the EEG feedback itself. Similarly, according to Logemann et al. (2010), non-specific factors may be responsible for the effects observed in healthy subjects. A more recent review by May et al. (2013) carried out on traumatic brain injury patients also concluded that positive therapeutic effects of the EEG-NFB could be achieved, however, all the reviewed studies were missing proper sham control groups (with pseudo-feedback based on an EEG signal recorded from another individual/another session, or an artificial signal generated by a computer) that would undergo fake EEG-NFB training. The same methodological weakness characterized the therapeutic-oriented ADHD studies analyzed in another review published recently by Arns group (Arns et al., 2014). The majority of the reviewed studies included a limited control group such as a semi-active control group (aiming to control non-specific effects like time spent interacting with a computer) or an active control group (comparison with a treatment with known therapeutic effects). Importantly, for all studies using proper sham-control groups the results of EEG-NFB training appeared to be negative. Controls for both clinical treatment and basic research of EEG-NFB training should include sham groups that account for factors such as spontaneous EEG changes, coach-subject interactions or attention effort that accompanies any EEG-NFB training. The effect of the independent variable (the specific EEG-NFB protocol) should be estimated as the measurement in the experimental group minus the same measurement in the sham-control group or alternatively in a group with a different protocol. Without such quantification the experimental results do not constitute evidence for the efficacy of the independent variable, i.e., feedback training.

Unfortunately, a sham-control for the positive effects is ethically challenging, since the use of placebo (which includes also sham groups), instead of clinical treatments, may lead to a deterioration of symptoms (Helsinki Declaration 1964). Thus, well-controlled EEG-NFB studies can only be carried out on treatment-resistant or healthy subjects.

The recent review series by Gruzelier (2013a,b, 2014) stemmed from the increasingly abundant number of studies devoted to EEG-NFB experiments performed on healthy participants. The author presented overwhelmingly positive interpretation concerning the state-of-the-art of EEG-NFB research, however these reviews included multiple studies which did not include proper controls for nonspecific training effects (as mentioned above).

Here, we selectively review only of those EEG-NFB studies which have included an appropriate control group(s) to quantitatively evaluate the effects of this type of training. The fundamental assumptions of EEG-NFB are based on the causal relationships between (1) the desired feedback signal and changes in brain activity, and (2) between induced EEG power changes and behavior. Therefore, we quantitatively assessed the efficacy of the protocols reported to induce changes in these two variables (EEG activity and behavior). We also examined any possible mutual relationship between these two measures. Our evaluation included quantitative estimations of the contribution of individual experimental factors to the final outcome. Each experiment that qualified for analysis was verified to determine whether EEG-NFB training induced a significant modulation of EEG and/or behavioral features, and whether the EEG modulation and behavioral changes occurred concomitantly.

## Methods

### Inclusion criteria

The research papers selected and reviewed in this study were identified by (i) a search of web resources (Google Scholar and PubMed) using the following keywords: biofeedback and EEG, neurofeedback and EEG, brain-computer interface and EEG, and EEG operant conditioning; and (ii) an examination of the reference lists of the retrieved articles. From the collected database we selected 86 articles that described experiments carried out using healthy adult participants.

### Two steps selection process

First, we selected articles which included separate control groups and those in which training was performed in two parallel groups, each group with a different EEG-NFB protocol. In latter case we assumed the two groups could serve as a control for one another (Bird et al., 1978; Allen et al., 2001; Keizer et al., 2010a). We included purely behavioral studies that lacked elaboration of the EEG data. In these studies the authors only used EEG for generating feedback signals, the EEG recordings obtained from within the training sessions or the pre- vs. post-training data were not analyzed and outcomes were only focused on the final behavioral effect of training.

This criterion identified eligible 40 publications that described 43 EEG-NFB experiments (a few articles described more than one experimental paradigm) that were performed on healthy volunteers (Table 1) and included different types of control groups: sham EEG-NFB, alternative type of activities (relaxation techniques, yoga), or no activity. We carefully evaluated all these experimental paradigms and in the second selection step we excluded studies in which control did not unequivocally address major nonspecific factors. In an illustrative example no activity was required from participants in the control group, not even attendance at the laboratory on a similar schedule. In this case, since the control group was not exposed to nonspecific factors that accompanied EEG-NFB training, it is not known whether the observed behavioral effects resulted from the treatment, or from factors such as regular tasks that require focused attention and/or trainer care. Similarly, the EEG spectrum could also depend on nonspecific behavioral training. Thus, for further analysis we only included experiments in which EEG's were recorded and analyzed in both EEG-NFB and control groups.

**Table 1:** **List of the analyzed studies (references in the second column) with their characteristics, including raw values of the experimental factors used for the analysis**.

	**Study**	**Protocol**	**Training intensity index**	**Number of participants**	**Average age**	**Modality**	**Electrodes position**
1	Allen et al., 2001	Alpha−	5.0	20	28	Auditory	T3, F7
2	Becerra et al., 2012	Theta−	15.0	7	65	Auditory	F4, C3, C3, P3, F7, F8
3	Berner et al., 2006	Beta1+	0.3	6	21	Auditory	Cz
4	Bird et al., 1978	Gamma+	8.0	11	23	Auditory	O1, O2
5	Bird et al., 1978	Gamma−	8.0	11	23	Auditory	O1, O2
6	Boxtel van et al., 2012	Alpha+	8.6	15	21	Auditory	C3 C4
7	Chisholm et al., 1977	Alpha+	1.0	12	21	Auditory	Oz
8	DeGood and Chisholm, 1977	Alpha+	1.0	10	20	Auditory	Pz
9	Egner et al., 2002	Alpha−/theta+	1.7	9	23	Auditory	Pz
10	Enriquez-Geppert et al., 2013	Theta+	10.0	16	25	Visual	Fz, FC1, FCz, FC2, Cz
11	Enriquez-Geppert et al., 2014	Theta+	8	19	24	Visual	Fz
12	Hoedlmoser et al., 2008	SMR+	10.0	16	24	Mixed	C3
13	Keizer et al., 2010a	Gamma+	7.0	8	23	Auditory	Oz, Fz
14	Keizer et al., 2010b	Gamma/beta−	6.1	7	22	Auditory	Oz
15	Kober et al., 2015	SMR+	7	10	24	Visual	Cz
16	Konareva, 2005	Alpha−/theta+	1.0	30	22	Auditory	C3/C4
17	Landers and Petruzzello, 1991	SCP+	0.2	8	no data	Visual	No data
18	Logemann et al., 2010	SMR+/theta−	5.3	14	21	Mixed	No data
19	Peeters et al., 2014	Alpha−	1	16	22	Visual	F3
20	Reichert et al., 2015	SMR+	10	28	45	Mixed	Cz
21	Reis et al., 2015	Alpha+	6	8	60	Visual	Fz
22	Reis et al., 2015	Theta+	6	8	60	Visual	Fz
23	Ring et al., 2015	Alpha−	1	12	23	Auditory	Fz
24	Ros et al., 2010	Alpha+	1.0	12	31	Visual	C3
25	Ros et al., 2010	Beta1+	1.0	12	31	Visual	C3
26	Ros et al., 2013	Alpha−	0.2	17	33	Visual	C4
27	Wang and Hsieh, 2013	Theta+	6.0	16	65	Mixed	Fz
28	Witte et al., 2013	SMR+/theta−	2.7	10	24	Visual	Cz

In a few articles the authors did not supply sufficient information regarding the experimental paradigm (denoted as ‘no data’ in the table). The “+” and “–” signs in the “Protocol” column denote the enhancement or suppression of particular frequency bands.

During this second selection step the following studies were excluded: (i) experiments that used EEG feedback but did not provide the results of the EEG analysis in the article; (ii) experiments that did not provide a description of the EEG results for the control group which precluded reliable analysis; (iii) experiments that did not engage the members of the control group in any type of activity (“non-intervention group”) and/or did not describe the control group manipulation); (iv) experiments that used non-responders (subjects not showing expected changes due to neurofeedback training) identified after completion of the training as a control. The identification of “non-responders” has been reported in published EEG-NFB experiments and clinical applications. It is possible that susceptibility to EEG-NFB training differs greatly between individuals, which alone demands further investigation (Weber et al., 2011; Wan et al., 2014; Nan et al., 2015). However, at present, it is not appropriate to restrict the experimental group to the participants with clear effects and exclude non-responders from the analyses. The set of papers selected for final analysis was composed of 28 experiments described in 25 studies (listed in Tables 1, **4**). Supplementary Table 1 lists the eliminated studies and reasons for exclusion.

### Key experimental factors

Many factors may potentially influence the success or failure of EEG-NFB procedures. Some factors can be controlled in our experimental paradigms, other cannot. An example of the latter is low training susceptibility of a subgroup of participants frequently referred to as non-responders. In order to identify potential non-responders and exclude them at the initial stages of screening for NFB training a new line of research has emerged focused on individual factors that might predict training success (Weber et al., 2011; Nan et al., 2015). Here we concentrate on the neurofeedback methodology, in which important factors influencing training success may be: feedback modality, training intensity, choice of EEG band(s) used for the feedback signal, and the number and positions of electrodes from which feedback signal is recorded. Since the role of these factors has not yet been quantified, we attempted to evaluate their influence in NFB training in the selected studies. Several other factors may also be important for EEG-NFB training, such as the age of the trainees, their personal traits and beliefs regarding EEG-NFB training (e.g., Witte et al., 2013), and trainer behavior; however, they could not be analyzed due to either insufficient variability of the data (e.g., with respect to age - in most cases the participants were university students) or a lack of a sufficient number of reports regarding a specific factor across the investigated experiments. In this study, we identified five factors which were investigated in a sufficient number of studies and had appropriate variability for statistical analyses in order to determine their influence on the training results.

Definitions used for all the analyzed factors were as follows:

- Protocol-EEG band or band combination used as the feedback signal;
- Number of bands-number of simultaneously manipulated EEG bands, i.e., amplitude of bands which were used to shape the feedback signal presented to the subject;
- Training Intensity index-training intensity was defined as the total number of training days divided by the average interval between them (in days). A higher Training Intensity Index indicated more intense training. The duration of a single training session was not taken into account;
- Feedback modality-modality of the sensory signal presented to the subject: visual, auditory, or visual and auditory;
- Number of EEG-NFB electrodes-number of electrodes used for feedback signal generation.

Since the factors listed above were differentially described in the individual studies, we applied common categorization scales for analyses. The range of these scales is presented in Table 2. The EEG bands, which were also differently specified in the reviewed publications, were grouped based on their frequency ranges to six unified categories defined in Table 3. Table 4 summarizes the parameter values for the database of all 28 experiments.

**Table 2:** **Assignment of the factors' row values to the categories used for *X*^2^and Kendall's T analyses**.

**Training intensity index**	**Feedback modality**	**Number of EEG-NFB electrodes**	**EEG-NFB electrode position**				
**Raw value**	**Category value**	**Raw value**	**Category value**	**Raw value**	**Category value**	**Raw value**	**Category value**
0–5	1	Auditory	1	1	1	Other than Cz	1
5–10	2	Visual	2	>1	2	Cz	2
>10	3	Visual and auditory	3				

**Table 3:** **Definition of the EEG frequency bands used in this review**.

**Band Name**	**Band width (Hz)**	**Comments**
SCP	0.5–2	Slow Cortical Potentials
Delta	2–4	
Theta	4–7	
Alpha	8–12	Also includes μ-rhythm (9–11 Hz)
Beta	12–30	Also includes SMR (12–15 Hz)
Gamma	31–100	

**Table 4:** **Success/Failure scores for studies (references in the second column) that qualified for analysis**.

**Number**	**Study**	**Protocol**	**EEG**	**Behavior**		
				**G**	**A**	**M**
1	Allen et al., 2001	Alpha	1	1		
2	Becerra et al., 2012	Theta−	0	1	0	0
3	Berner et al., 2006	Beta1+	0	0		0
4	Bird et al., 1978	Gamma+	1			
5	Bird et al., 1978	Gamma−	0			
6	Boxtel van et al., 2012	Alpha+	1	0		
7	Chisholm et al., 1977	Alpha+	1	0		
8	DeGood and Chisholm, 1977	Alpha+	0			
9	Egner et al., 2002	Alpha-/theta+	0	0		
10	Enriquez-Geppert et al., 2013	Theta+	1	0	0	0
11	Enriquez-Geppert et al., 2014	Theta+	1	1		1
12	Hoedlmoser et al., 2008	SMR+	1	1		1
13	Keizer et al., 2010a	Gamma+	1	1	1	
14	Keizer et al., 2010b	Gamma/beta−	1			
15	Kober et al., 2015	SMR+	1	1	1	1
16	Konareva, 2005	Alpha-/theta+	0			
17	Landers and Petruzzello, 1991	SCP+	0	1		
18	Logemann et al., 2010	SMR+/theta−	0	0	0	
19	Peeters et al., 2014	SMR+	0			
20	Reichert et al., 2015	Alpha−	1	0		
21	Reis et al., 2015	Alpha+	1	1		1
22	Reis et al., 2015	Theta+	1	1		1
23	Ring et al., 2015	Alpha−	1	0		
24	Ros et al., 2010	Alpha−	1	0		
25	Ros et al., 2010	Beta1+	0	0		
26	Ros et al., 2013	Alpha−	1			
27	Wang and Hsieh, 2013	Theta+	1	1	1	1
28	Witte et al., 2013	SMR+/theta−	0			

Training results: 1, training success; 0, training failure. “EEG” column lists the results on the modulation of EEG features, “Behavior” column contains the list results in the behavioral domain, G, general effects of the training obtained in any of the investigated behaviors; A, attention; M, memory. Values in column G may also include effects not classified to attention (A) and memory (M) groups.

### Selection of thematic groups

**Figure 1:**
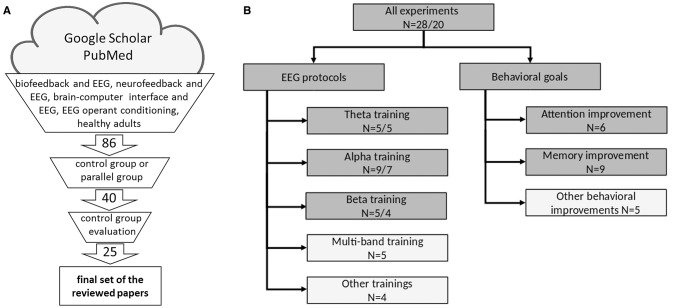
**(A)** Schematic representation for article selection. **(B)** Schematic representation of the experiments used in the review. Dark gray boxes specify the experimental paradigms grouped for analytical studies according to EEG (left branch) or behavioral protocol (right), light gray boxes specify groups not included in the analyses. In the EEG protocols branch, N denotes the number of experiments included in each EEG and behavioral group (EEG/behavioral) where EEG effects (changes of the EEG spectrum) were analyzed. In the Behavioral goals branch N denotes the number of experiments in each behavioral or cognitive group. The 28 EEG-NFB experiments were used for analyses. Of these studies, smaller groups were defined according to their specific EEG training protocols or the expected behavioral/cognitive effects. The groups are not mutually exclusive; both types of data were often analyzed in the same study, which resulted in multiple classifications. Some experiments also investigated more than one behavioral goal (Table 4). Protocols that could not be attributed to any of the specific subgroups (lowermost boxes “Other”) contributed only to the general analysis.

To assess the effectiveness of various EEG-NFB protocols and address the issue of the large diversity of applied protocols and investigated putative behavioral effects, we divided the database into groups based on the (i) EEG features used for training purposes and (ii) the behavioral tasks administered to the subjects (Figure 1). We then analyzed the data to identify the correlations between training factors (defined in paragraph 2.2.) and the observed EEG modulation and/or behavioral changes.

**Figure 2:**
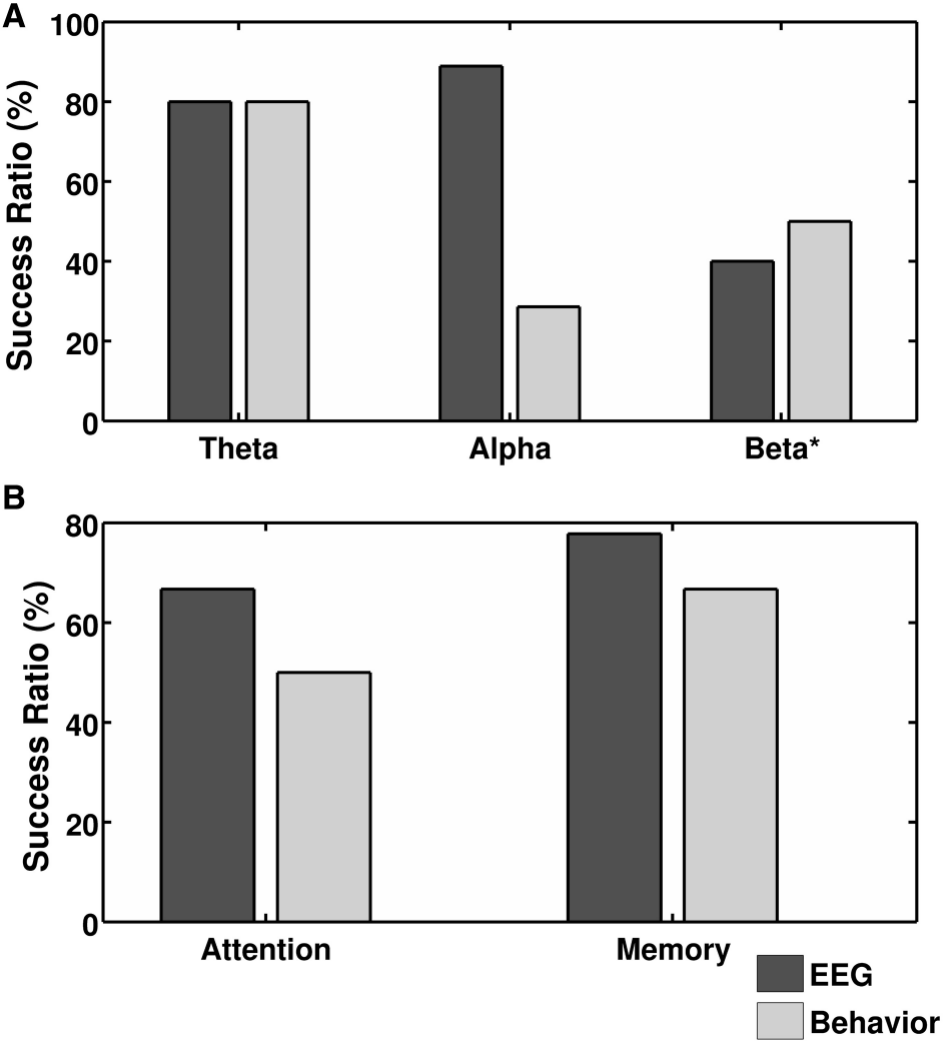
**Success ratio of the spectral EEG changes (dark gray bars) and behavioral improvement (light gray bars) calculated for: (A) experiments grouped according to training with particular frequency band protocols and (B) training aimed at different behavioral effects (i.e., improvement in attention and memory)**. Only protocols used in five or more experiments are included in the graphs with exception to four behavioral experiments in the Beta Group designated by ^*^.

- We selected three groups based on the frequency bands–theta (4–8 Hz), alpha (8–12 Hz) and beta1 (12–20 Hz), with a minimum of *n* = 5 experiments. Both, the up- or down-regulation of the band (as defined in Table 3) or its fraction were included in the same protocol group (i.e., the up-regulation of “low alpha” and the down-regulation of “high alpha” were both considered alpha protocols aimed at the assessment of the alpha band protocol effectiveness). This approach enables the estimation of whether training based on a particular frequency band induces the desired changes in the EEG or behavioral domains. For other single and multi-band protocols, we did not find a sufficient number of experiments for statistical evaluation.
- Within the behavior-oriented experiments, we also identified two groups (based on the behavioral tests described): An Attention Group: experiments targeted to change performance measured by attention tests (e.g., TOVA–Test of Variables of Attention, SSRT–Stop Signal Reaction Times, CPT–Continuous Performance Test or others) and a Memory Group: studies aimed at the identification of any type of memory improvement (estimated by different types of recall and recognition tasks; Figure 2).

### Definition of successful training

In the reviewed articles, the efficacy of EEG-NFB training in the induction of EEG modulation was typically evaluated by comparing the amplitudes (absolute or relative) of the monitored EEG bands (in the case of single band protocols) or the amplitude ratios between the manipulated bands (in the case of multi-band protocols, i.e., theta/alpha or SMR/theta ratios) recorded before and after the whole training (interpreted as delayed effects of neurofeedback or, more general, brain plasticity [50]) or within/between training sessions. Significant differences of these parameters between the experimental and control groups indicated successful training in the EEG domain. The changes in behavioral performance were evaluated based on tests or measures typical for the given type of activity.

We reviewed each article and individually qualified the training results as a failure (0) or success (1) based on the statistical measures used by authors. Training was considered as being a success:

- If the measured effect (EEG or behavioral) was significantly different (at the level of *p* < 0.05 for any type of statistics) from the result achieved in the control group;
- If the group effect or interaction between the groups and time were significant in an ANOVA test;
- In cases where direct statistical comparison of the experimental and control groups were not available in the original text, we qualified them (Table 4) as a success when the comparison between the pre- and post-training measurements provided a different outcome in the experimental and control groups (e.g., a significant change in the experimental group and no change in the control group).

If direct (i) or (ii) and indirect (iii) comparisons for EEG or behavior were not described in the articles, we qualified the outcome of the training as giving no results. We considered EEG-NFB training as successful if either EEG or behavioral change reached significance. Similarly, a study was defined as a success when one of several results available for one category (e.g., scores from several attention tests or an amplitude change of different EEG bands) was unambiguously described as significantly changed as a result of the training.

### Methods of statistical analyses

We estimated the correlation of each training factor with the EEG and behavioral success scores for all experiments and specific groups (defined in the paragraph' Selection of thematic subgroups'). An analysis was performed only for groups that comprised five or more experiments. For consistent comparison of all factors between all experimental groups, we used two non-parametric tests: *X*^2^ to test for independence of training outcome and the Kendall's T (tau) rank correlation coefficients to precisely assess the strength and direction of any significant association. Although Kendall's T coefficient is similar to the Spearman rank correlation statistic, which is used to measure rank correlation, the advantage of using Kendall's T is in the direct interpretation of the probability of observing concordant and discordant pairs (Hauke and Kossowski, 2011).

The partial η^2^ (Bakeman, 2005; Lakens, 2013) can be used to estimate effect size from published studies using information about F statistic provided by the authors. It is suitable for the results extracted from multiple experiments with similar design and testing one (or equivalent) dependent variable. Unfortunately, the designs used in the reviewed papers did not meet these criteria. First of all, there was a great variety of EEG features (dependent variables) that underwent treatment. Next, within the same trained EEG feature, the statistics were available either for within group or between group analyses. Often, the statistics were incompletely described, which made calculation of effect size impossible. The details of statistical results provided in the reviewed articles are presented in the Supplementary Table 2.

After grouping the available effect sizes according to the training protocol and source of the effect (within group, between groups, interaction) the groups were not numerous enough for quantitative comparisons. We managed to construct only one group of 8 reports regarding alpha training from which we could extract F statistics for within subject effects which enabled evaluation of unspecific changes of this band.

Since the calculation of statistical power for all the other training factors was not possible, we used a more gross, binary approach. Instead of using F values we qualified experiments as a failure (0) or success (1, Section Definition of successful training) and calculated a success ratio (SR) defined as the percentage of successful studies within the total number of studies of the given set.

## Results

### General characteristics of the participants

The average age of the participants ranged from 20 to 65 years, with median = 24 (most participants were students) and mean = 30 ± 14. The difference between the mean and median age was caused by two experiments in which the participants' ages ranged between 55 and 75 years (6, 41 in Table 1, correspondingly).

The mean number of subjects who participated in a single experiment was 26 ± 10, with an average experimental group size of 13 ± 6 subjects. The diversity of the relatively small number of qualified experiments permitted selection of two behavioral groups: Attention (*n* = 6) and Memory (*n* = 9) and three EEG protocol groups: Theta (*n* = 5), Alpha (*n* = 9), and Beta (*n* = 5) fulfilling the criterion of minimum group size. Seven out of nine experiments in the Alpha Group, four out of five in Beta Group and all five Theta Group experiments included also behavioral tests.

### EEG-NFB training methods (EEG protocols, training intensity, and signal modality)

Most of the experiments (23 of 28) used single EEG band protocols for EEG-NFB training, and most of these (17) intended to up-regulate the amplitudes of the trained band: theta (*n* = 4), alpha (*n* = 5), beta (including SMR, *n* = 5), gamma (*n* = 2), or slow cortical potentials (SCP, *n* = 1). The remaining five experiments (18%) used multi-band protocols that aimed to change the ratio of the amplitudes of the employed bands (Table 1).

The Memory Group (*n* = 9) was composed of experiments using only single band protocols: five experiments had theta protocol, three beta and one alpha. In the Attention Group (*n* = 6), the majority of experiments used a theta protocol (*n* = 3), and the three remaining studies used beta, gamma and SMR/theta protocols.

The daily training sessions across all experiments typically consisted of multiple, few minutes long runs interrupted by short pauses. The average training was 7.7 ± 3.8 sessions with 3 ± 2.4 day intervals; thus, the training intensity was relatively low compared with EEG-NFB training arrangements in clinical practice [4]. The average Training Intensity Index was 5.5 ± 3.4 for all experiments. In 46% of the experiments the EEG-NFB modality was auditory, in 40%, it was visual, and in 14%, it was mixed (visual and auditory).

### Location of electrodes

In the two behaviorally oriented groups (Attention and Memory) we did not find any consistency in the placement of feedback electrodes. Out of six experiments, oriented on training of attention, five different electrode(s) locations were used. In the Memory Group in five experiments the electrodes were placed at Fz or included this location, in the remaining four experiments we did not note any consistency in the placement of electrodes.

Among the EEG protocols most consistent electrode placement was observed in the Theta Group where all single electrode setups used the Fz location and one of two multi-electrode setups used frontal locations including Fz. All experiments with training provided from Fz solely or together with other locations succeeded in evoking EEG modulation (seven experiments, four using theta protocol, two using alpha protocol and one using gamma protocol). In the Beta Group four experiments used mainly electrodes located over central sulcus (in the three experiments electrodes were positioned at Cz and in two in C3 locations according to the 10–20 international system) and one in the F3 position. In the Alpha Group the most commonly used locations were frontal (Fz, F3, F7) and central (C3, C4), one experiment used occipital (Oz) and one parietal (Pz) location.

### Dependence of successful training on the analyzed factors

According to the descriptions provided by the authors, EEG-NFB training yielded significant results in the EEG domain for 17 out of total 28 experiments which resulted in a 60.7% success ratio (SR) and in 10 out of 20 behaviorally-oriented experiments, which provided a SR of 50 %. SR values are given in Table 4. We did not find a significant relationship between behavioral or EEG success in general (*X*^2^ = 0.91, *p* = 0.33) or any of the considered training factors (*X*^2^ and T statistics for all analyzed factors are given in Table 5) except for number of bands which negatively correlated with the resulting EEG changes (*X*^2^ = 4.2303, *p* = 0.0397; *T* = −0.3887, *p* = 0.0472). We also observed a tentative relation between SR and training intensity index (TI). In a sample of 6 studies, which had at least four training sessions completed on consecutive days (excluding weekends) SR was 100%.

**Table 5:** **Dependence (*X*^2^) and correlation (T) values for EEG training success and analyzed factors**.

	**Number of bands**	**Training Intensity**	**Number of participants**	**Stimulus modality**	**Number of electrodes**
*X*^2^	4.2303	15.9216	10.9778	0.5865	2.9365
P for *X*^2^statistics	0.0397	0.2534	0.6878	0.7458	0.4015
T	−0.3887	0.3145	0.2932	0.1087	0.0304
P for T statistics	0.0472	0.0606	0.0902	0.5886	0.8966

Correlation was considered significant when p < 0.05 for both X^2^ and Kendall T correlation.

In the consecutive steps of our review we analyzed the effectiveness of training for experiments classified to smaller groups specified by EEG or behavioral protocols.

### Efficacy of employed EEG protocols

Theta and alpha EEG band appeared to be highly susceptible to EEG-NFB procedures resulting in SR's of 80 and 89% respectively, trainings targeted at the beta band were less effective with a SR of 40%. The experiments using multi-band protocols were very diverse and only one of five yielded changes in EEG spectra, which is in line with our general conclusion of negative relation between number of bands used for training and induced EEG changes. In the behavioral domain out of three investigated protocols only theta was highly effective at inducing behavioral changes with a SR of 80%, the least effective was alpha protocol with a SR of 28% (Figure 2).

### Efficacy of the training aimed at specific behavioral effects

The SR of the desired behavioral effects in Attention and Memory Groups reached 50 and 67%, respectively (Figure 2). The effectiveness of inducing EEG changes was even higher with an SR of 67% (attention) and 78% (memory). A comparison of the chosen experiments that had positive results in the EEG domain and included behavioral tests before and after the training did not reveal any frequency band that was specifically effective for increased performance in attention or memory tests. In the Memory Group, of six experiments which yielded positive training results in both domains three experiments used theta, two SMR and one alpha protocol, additionally one theta protocol resulted with reported lack of significant results in the behavioral domain. In the Attention Group, there were four eligible experiments in which three cases had positive results in both EEG and behavioral domains, but each of them had a different protocol.

### Summary of the efficacy of employed EEG protocols

Our analysis demonstrated: (i) the effectiveness of the theta and alpha protocols at inducing EEG modulations; (ii) a negative correlation between the number of trained bands and success in evoking EEG modulations, (iii) a lack of specificity of the popular EEG-NFB protocols in obtaining desired behavioral changes.

## Discussion

The theoretical basis for EEG-NFB is that brain activity, modified according to the targeted changes of the EEG signal, can cause a plastic reorganization within the involved brain networks and lead to an expected improvement in a specific behavioral task (Anguera et al., 2013). The findings of this review cannot unambiguously disprove or support this hypothesis, since behavioral effects were not convincingly validated by any specific training protocol. This ambiguity may results from a limited number and high diversity of the available descriptions of well-controlled NFB studies.

The scientific community has produced a multitude of articles with enthusiastic case reports on EEG-NFB training. However, a rigorous scientific approach to EEG-NFB is rare, and experiments performed on healthy participants to study the effectiveness and/or mechanism of training are very limited; we identified only 86 relevant reports. From this list we selected 43 EEG-NFB experiments which described control groups. We next assessed the quality of the control paradigms and excluded all experiments that did not allow for unambiguous attribution of the results to the effects of EEG-NFB training.

The final, small number of accepted studies (*n* = 28) is alarming because it suggests that the field of EEG-NFB research is dominated by poorly controlled experiments. Analogous conclusions were drawn in other quantitative reviews of EEG-NFB research. In her review, Niv (2013) attempted to assess the efficacy of EEG-NFB applied to patients with neurological disorders (e.g., ADHD, autism, epilepsy) and identified only 22 well-controlled experiments. The review concluded that “only few controlled studies exist and more and better-organized research is necessary to confirm the efficacy and effectiveness of neurofeedback”. The two meta-analyses of Arns et al. (2009) and Lofthouse et al. (2012), who investigated the efficacy of EEG-NFB treatment applied to children diagnosed with ADHD, included only 15 and 14 studies, respectively.

In our analysis of 28 well-controlled studies, we calculated the SR separately for EEG (60.7%) and behavioral (50%) effects. These numbers demonstrated that EEG-NFB training could influence both the amplitude of the chosen EEG signal and behavior. Because the effects were not consistent, it is essential to carefully evaluate all factors of experimental design to select those that promote successful training (see also review by Vernon et al., 2009). The fact that most of the investigated factors did not correlate with training success is worth consideration itself. The intuitively important factors such as feedback modality did not affect training results. SR for auditory and visual paradigms were similar (53% and 63%, respectively). Training success did show not dependency on the intensity of the stimuli (*X*^2^ = 15.9, *p* = 0.25). High SR (75%) averaged from four experiments using mixed modality stimuli should be treated with caution, however Vernon et al. (2004) in his review suggested the optimal results with combined, auditory and visual feedback.

The assessment of the SR in the selected experiments was possible for theta, alpha and beta bands. From those three protocols the most effective in changing the EEG spectra were training involving the theta or alpha band. Alpha training calls for special attention for its popularity despite the low behavioral SR (out of nine well controlled EEG-NFB studies with alpha training, seven aimed to induce behavioral improvement, and only two were successful (Allen et al., 2001–reported mood increase and Reis et al., 2015–reported improvement of memory; comp. Table 4). It seems that the alpha band is highly susceptible to various manipulation. This notion is supported by Williams (1977) who used two sham-feedback groups (with no real experimental group) and reported that an increase in alpha band amplitude was achieved only in subjects who had been told that they were involved in the real alpha-inducing experiments. The subjects who were informed that they participated in the control group did not develop this change. Quandt et al. (2012) induced changes in the upper alpha and beta bands in subjects who observed actions performed by other individuals, further supporting the high susceptibility of the alpha band to nonspecific manipulations. Finally, Dempster and Vernon (2009) showed that an increase in alpha band amplitude often observed during EEG-NFB trainings might merely represent return to baseline level disturbed by training procedure. This rebound might be enhanced in EEG-NFB groups and impaired when subjects are engaged in e.g., sham feedback. Thus, inclusion of baseline measurements in scientific reports might be a valuable addition in studying inferences about EEG-NFB effects on EEG. In 4 out of 8 reviewed studies, alpha was found to be changing in both experimental and control conditions. In order to estimate non-specific alpha changes we pooled together increases and decreases of alpha over both sessions and epochs in all experimental conditions, resulting in average effect size 0.32 (STD = 0.15) (positions 6, 8, 9, 11, 31, 32, 34, 36 in Supplementary Table). The high susceptibility of the alpha band to neurofeedback manipulation, as resulting from our review, was, however, not accompanied by any specific behavioral effects.

There is an assumption behind NFB practice concerning specific protocols that target particular behavioral goals (Egner and Gruzelier, 2004; Keizer et al., 2010b; Gruzelier, 2013a,b; Enriquez-Geppert et al., 2014). However, our analysis of the training protocols that aimed to improve attention or memory performance does not enable such attribution of any of the frequency bands used for training to these two behavioral goals showing no privilege of any specific frequency band. A similar observation was reported by Arns et al. (2009) in conclusion of their meta-analysis: while EEG-NFB therapy provided, on average, positive therapeutic effects for children with ADHD, there was no difference in the effect size for different EEG-NFB protocols (SCP [0.5–2 Hz], SMR [12–15 Hz]/theta and beta/theta).

This lack of specificity between the frequency band of an EEG-NFB protocol and its behavioral effect does not necessarily discount the basic concept of behavioral correlates of specific EEG frequencies (Anguera et al., 2013). When investigating the roots of inconsistent EEG-NFB effects, a more careful examination of the old issue of spatial resolution and source localization in EEG recording is warranted (Zuberer et al., 2015). The recorded EEG signal can be generated by an infinite number of different sets of current sources, even with the assumption of an infinite number of recording electrodes (Fender, 1987). In the case of EEG-NFB, where it is expected to modify the activity of the local networks (Wood and Kober, 2014), the signal collected by a few EEG-NFB electrodes is generated also by other brain regions than those desired as a training target (Witte et al., 2013). A partial solution to this problem could be high resolution EEG combined with EEG source localization methods to enable training focused on specific regions. Although, results of preliminary studies, using multi-electrode EEG-NFB such as LORETA-EEG-NFB, Z-score-EEG-NFB, or blind source separation, seem promising (Cannon et al., 2006, 2009; Koberda et al., 2012; Bauer and Pllana, 2014; White et al., 2014) more experiments with sham control groups are necessary to verify this approach as a valuable EEG-NFB tool. The source localization problem worsens in experiments where a single EEG-NFB electrode is used. Unfortunately, a single electrode approach is the most common practice in EEG-NFB training and therapy. We argue that such a protocol would not be able to precisely affect the putative regions responsible for targeted behavior.

It is important to underline that several thoroughly prepared experiments clearly show positive effects of EEG-NFB training (Allen et al., 2001; Hoedlmoser et al., 2008; Keizer et al., 2010a). However, in other (equally thorough) studies, EEG-NFB was not found to be successful (Egner et al., 2002; Berner et al., 2006; Logemann et al., 2010). As noted above, this discrepancy might stem from the lack of optimal training paradigms. The presented quantitative review offers suggestions for concrete modifications of the used paradigms, which might improve the results of EEG-NFB training. We subsequently discuss two particular factors that originate from experiments collected in this review.

### The don'ts: the number of EEG bands used for training

One of our findings, is the negative correlation between the number of bands used to compose the feedback signal and the success of training, i.e., complicated protocols which promoted and/or inhibited several bands worsen the final results. This may be related to the interdependence of the EEG frequencies, i.e., their susceptibility to follow the modulation of the other, most often neighboring frequencies. Band interdependence has been reported by Ros et al. (2013) who demonstrated that training directed for up-regulation of the alpha band was accompanied by changes in the flanking frequencies (theta and beta bands). The interdependence of EEG frequencies may also affect the expected behavioral changes because the modulation of neighboring bands trained in opposite directions may cancel out the effects of up-regulation of the targeted band. Similarly, the observed behavioral change may result from an up-regulation of bands neighboring the band that was intentionally trained. This situation might at least partially explain why so many authors observed positive behavioral results despite the lack of noticeable modifications of the trained EEG band.

However, the issue requires deeper investigation as there is a contradictory example of theta band amplitude increase not accompanied by an increase in the flanking alpha frequency amplitude (Enriquez-Geppert et al., 2013).

### The do's: location of EEG-NFB electrodes

The analysis of experiments from the Theta Group showed that neurofeedback mediated by the electrodes positioned above well-identified sources of a given EEG frequency (e.g., frontal regions in case of frontal-middline theta training) might bring expected changes in the EEG spectrum (Keizer et al., 2010a; Boxtel van et al., 2012; Wang and Hsieh, 2013). In general, however, the targeted frequencies are generated by many structures of dispersed neural networks and the use of a one or two, arbitrarily located EEG-NFB electrodes (and they reference), does not allow for non-ambiguous estimation of the brain sources involved. A way to partially mitigate this issue might be the use of multiple electrodes located in the appropriate position for capturing activity of the brain regions associated with the targeted behavioral functions. Indeed, the rare experiments in which electrode location was justified for physiological reasons appeared to be successful (Enriquez-Geppert et al., 2013, 2014), which likely results from a substantial contribution of relevant sources into feedback signals.

Suitably positioned multiple electrodes increase the chances of collecting feedback signals from many regions engaged in the target activity. Attention is a cognitive function with relatively well-known networks of involved brain structures and a popular goal for improvement in many EEG-NFB paradigms. The fronto-parietal network (Buschman and Miller, 2007) is one of the brain systems that support attentional mechanisms and it is not surprising that most protocols that successfully increased attention used feedback signals from several EEG-NFB electrodes located at frontal and parietal locations (Allen et al., 2001; Becerra et al., 2012; Enriquez-Geppert et al., 2013), or from a single electrode located over frontal areas (Vernon et al., 2004). In contrast, an experiment with a similar goal, which used C3/C4 electrodes (Boxtel van et al., 2012) to record feedback signals did not achieve significant behavioral changes after EEG-NFB training. We posit that the locations of the EEG-NFB electrodes in many experiments reviewed in this analysis were non-optimally placed for an expected result. It is, therefore, important that future EEG-NFB experiments should identify optimal training electrode locations based on existing knowledge regarding the anatomical and functional substrates of various behavioral tasks. It appears feasible that for a dispersed target network new training paradigms should be developed in which the feedback signals would be weighed by a component analysis from many recording electrodes.

## Conclusions

We posit that neurofeedback methodology used in the majority of the reviewed experiments did not enable proper targeting of the brain regions responsible for control over the desired behavioral changes. This may explain the lack of correlation between the changes induced in the trained EEG signal and the modification of the targeted behavior, as well as lack of correlation between the remaining analyzed factors and training success. We, therefore, recommend the following factors for improved EEG-NFB training efficacy:

-Use single band protocols.
-Localize brain regions functionally correlated with the targeted behavior for optimal electrode placement (if possible).
-Use of viable EEG source localization methods for the feedback signals used in the training (if possible).

## Author contributions

JR Review concept, data analyses, main conclusions, article drafting. KJ Data analyses, verification of analytical methods, proof reading. KP Data analyses, proof reading. EK Conclusions verification, data analyses, proof reading. RC Data analyses, verification of analytical methods, proof reading. AW Verification of the review concept and of the conclusions.

### Conflict of interest statement

The authors declare that the research was conducted in the absence of any commercial or financial relationships that could be construed as a potential conflict of interest.
