# Supplementary material for: The Do's and Don'ts of Neurofeedback Training: A Review of the Controlled Studies Using Healthy Adults
Source: Front Hum Neurosci. 2016 Jun 17;10:301. doi: 10.3389/fnhum.2016.00301 (PMC4911408; doi:10.3389/fnhum.2016.00301)
Supplement: Supplementary file 1 [file Table1.docx]

Table Suppl. I. Studies excluded by the second selection criteria.
The last column describes the rationale behind the exclusion:

A - no EEG results available;

B - no EEG results available for control group, no comparison of EEG results between the experimental and the control group;

C - non-intervention control group, or control group not described;

D - control group defined on the basis of the training outcome (composed from non-responders).

|  | **Reference** | **Exclusion justification** |
| --- | --- | --- |
| 1 | Escolano et al., 2011 | B, C, additionally, the EEG results for experimental group were analyzed after exclusion of non-responders. |
| 2 | Ghaziri et al., 2013 | A |
| 3 | Gruzelier et al., 2013c | B |
| 4 | Gruzelier et al., 2014b | B, C |
| 5 | Hanslmayr et al., 2005 | D |
| 6 | Lecomte, 2011 | B |
| 7 | Mohr et al., 1998 | D |
| 8 | Nan et al., 2012 | C |
| 9 | Paul et al., 2011 | C |
| 10 | Raymond et al., 2005 | B |
| 11 | Reiner et al., 2014 | B |
| 12 | Rostami et al., 2012 | B, C |
| 13 | Strizhkova et al., 2012 | C, no description of NFB training paradigm |
| 14 | Vernon et al., 2003 | The article described two experiments; in our analysis one of them was used as the control group for the other one (beta/theta) and consequently not considered as a separate study. |
| 15 | Zoefel et al., 2011 | C, additionally, the EEG results for experimental group were presented after exclusion of non-responders. |

Table Suppl. II. Partial eta-squared effect size values of investigated studies (A – EEG results, B – behavioral results) . # - Studies are identified by the numbers from Tables 1 and 4.

A. Eta-squared values for EEG effects

| # | **Study** | **Protocol** | **F (df)** | **Eta** | **Comments** |
| --- | --- | --- | --- | --- | --- |
| 1 | Allen et al., 2001 | Alpha Hemispheric asymmetry | 2.58(4,64) | 0.14 | Interaction (trained hemisphere X training session) |
| 2 | Becerra et al., 2012 | Theta- | - | - | No degrees of freedom information |
| 3 | Berner et al., 2006 | Beta1+ | 1.94(12,108) | 0.18 | Interaction (condition X epoch X electrode) |
| 4 | Bird et al., 1978 | 40 Hz+ | 3.17 (7,84) | 0.21 | Within (Increase in 40Hz EEG over training sessions) |
| 4 | Bird et al., 1978 | 40 Hz+ | 2.56(7,84) | 0.18 | Within (Increase in beta over training sessions) |
| 5 | Boxtel et al., 2012 | Alpha+ / Beta+ / Music relaxation | 66.96(2,90) | 0.48 | Within (Increase in alpha over training session for all conditions) |
| 5 | Boxtel et,. al 2012 | Alpha+ / Beta+ / Music relaxation | 13.99 (2,90) | 0.23 | Within (decrease of beta over training sessions for all conditions) |
| 6 | Chisholm et al., 1977 | Alpha+ | 2.26(7,77) | 0.17 | Within (Alpha increase in NFB condition) |
| 6 | Chisholm et al., 1977 | Alpha+ | 2.84(7,77) | 0.21 | Within (Alpha decrease in Control condition) |
| 6 | Chisholm et al., 1977 | Alpha+ | 2.17(14,23) | 0.57 | Interaction (condition X epoch) |
| 7 | DeGood and Chisholm, 1977 | Alpha+ | 9.85(1,18) | 0.35 | Within (Alpha change for all conditions) |
| 7 | DeGood and Chisholm, 1977 | Alpha+ | 16.28(1,18) | 0.48 | Main (alpha resting state before/after training change for all conditions) |
| 8 | Egner et al., 2002 | Alpha/theta ratio | 3.27(1,16) | 0.17 | Main (NFB X Control alpha/theta ratio difference between epochs) |
| 8 | Egner et al., 2002 | Alpha/theta ratio | 2.36(4,13) | 0.42 | Interaction (condition X epoch) |
| 8 | Egner et al., 2002 | Alpha/theta ratio | 2.83(1,16) | 0.15 | Main (NFB X Control alpha/theta ratio difference between training sessions) |
| 8 | Egner et al., 2002 | Alpha/theta ratio | 2.06(4,13) | 0.38 | Interaction (condition X training session) |
| 9 | Enriquez-Geppert et al., 2013 | Theta+ | 6.67(1,29) | 0.19 | Main (theta difference between conditions) |
| 10 | Enriquez-Geppert et al. 2014 | Theta+ | 6.23(6,39) | 0.50 | Main (alpha difference between conditions) |
| 11 | Hoedlmoser et al., 2008 | SMR+ | 7.81(1,23) | 0.25 | Interaction (condition X epoch) |
| 12 | Keizer et al., 2010a | Gamma+ | 11(2,15) | 0.59 | Interaction (condition X training session X epoch) |
| 12 | Keizer et al., 2010a | Gamma+ | 6.6(2,15) | 0.47 | Interaction (condition X training session) |
| 13 | Keizer et al., 2010b | Gamma/beta- | 14.2(1,13) | 0.59 | Interaction (condition X training session) |
| 14 | Kober et al., 2015 | SMR+ | 48.98(1,5) | 0.91 | Regression model |
| 15 | Landers and Petruzzello, 1991 | Slow Potential feedback | 5.22(2,21) | 0.33 | Main (Increase in 13-30 Hz EEG in right hemisphere feedback con) |
| 16 | Logemann et al., 2010 | SMR+/theta- |  |  | No significant effects |
| 17 | Reichert et al., 2015 | Alpha- | 8.56(1,26) | 0.25 | Main (responders vs. non-responders) |
| 18 | Reis et al., 2015 | Alpha+ |  |  | Only p-values presented |
| 18 | Reis et al., 2015 | Theta+ |  |  | Only p-values presented |
| 19 | Ring et al., 2015 | Alpha- | 11.97 – 14.02 (1,22) | 0.35 - 0.39 | Within (change in alpha over session at all electrodes in all conditions) |
| 19 | Ring et al., 2015 | Alpha- | 4.08 – 5.52 (4.88) | 0.16-0.20 | Within (change in alpha over epoch at all electrodes in all conditions) |
| 19 | Ring et al., 2015 | Alpha- | 6.35 -10. 77(1,22) | 0.22 -0.33 | Interaction (condition X training session at all electrodes) |
| 20 | Ros et al., 2010 | Alpha - | 2.7(10,110) | 0.20 | Within (Alpha decrease over epochs in trained hemisphere) |
| 20 | Ros et al., 2010 | Beta1+ | 1.7(10,110) | 0.13 | Not significant  Within (Beta decrease over epochs) |
| 21 | Ros et al., 2013 | Alpha- | 2(11,352) | 0.06 | Within (Effect of epoch on alpha level) |
| 21 | Ros et al., 2013 | Alpha- | 2.0(11,351) |  | Interaction (Condition X epoch) |
| 22 | Wang and Hsieh, 2013 | Theta+ | 57.07(1,28) | 0.67 | Main (Difference between conditions) |
| 22 | Wang and Hsieh, 2013 | Theta+ | 8.11(11,308) | 0.23 | Within (Increase in theta over session in the Old (ONFT) subjects condition) |
| 23 | Wang and Hsieh, 2013 | Theta+ | 21.81(11,308) | 0,44 | Within (Increase in theta over session in the Young (YNFT) subjects condition) |
| 23 | Witte et al., 2013 | SMR+ | 4.51(1,18) | 0.20 | Within (effect of epoch on SMR level ) |
| 23 | Witte et al., 2013 | SMR+ | p <0.01  R^2^ = 0.22 | Slope =0.023 | Linear regression (epoch X SMR level in NFB condition) |
| 23 | Witte et al., 2013 | SMR+ | p = 0.35  R^2^ = 0.78 | Slope =0.008 | Linear regression (epoch X SMR level in Control condition) |

B. Eta squared values for behavioral effects.

| # | **Study** | **Protocol** | **F (df)** | **Eta** | **Comments** |
| --- | --- | --- | --- | --- | --- |
| 1 | Allen et al., 2001 | Alpha Hemispheric asymmetry | 4.56(6,96) | 0.22 | Interaction (trained hemisphere X reported emotion) |
| 2 | Berner et al., 2006 | 40 Hz + / Beta + | 0.03(1,10) | 0.003 | Within (Non-significant,  training effects on memory performance) |
| 3 | Boxtel et al., 2012 | Alpha+ / Beta+ / Music relaxation | 0.07(2,47) | 0.003 | Within (Non-significant,  effect on reported mood (strongest effect from all reported)) |
| 4 | Chisholm et al., 1977 | Alpha + | 11.14(1,11) | 0.50 | Within (decrease of situational reactivity in sham condition) |
| 4 | Chisholm et al., 1977 | Alpha + | 12.71(1,11) | 0.54 | Within (decrease of situational reactivity in music control condition) |
| 5 | Hoedlmoser et al., 2008 | SMR+ | t(13) = -3.49 | 0.48 | Within (Increase if sleep spindle number for nfb condition only) |
| 5 | Hoedlmoser et al., 2008 | SMR+ | t(15) = 3.169 | 0.40 | Within (decrease of sleep onset latency for nfb condition only) |
| 5 | Hoedlmoser et al., 2008 | SMR+ | t(8) = -3.281 | 0.57 | Within (Increase in RET_1_ scores for nfb condition only) |
| 5 | Hoedlmoser et al., 2008 | SMR+ | t(8) = -2.956 | 0.52 | Within (Increase in RET_2_ scores for nfb condition only) * Post-hoc for non-significant interaction test |
| 6 | Keizer et al., 2010a | Gamma +/- | 10.57 (1,11) | 0.49 | Interaction (training X shape repetition X location repetition) |
| 6 | Keizer et al., 2010a | Gamma + | pearson r = 0.82,  p < 0.05 | 0.82 | Correlation between percent change in intelligence test and percent change in gamma power in gamma + condition. |
| 7 | Kober et al., 2015 | SMR+ | t(9) = -2.78 | 0.46 | Within (increase of vvm2 score only in experimental condition) |
| 8 | Landers and Petruzzello, 1991 | Slow Potential feedback | 11.97(1,7) | 0.63 | Within (decrease of shooting performance in sham feedback condition) |
| 8 | Landers and Petruzzello, 1991 | Slow Potential feedback | 4.97(1,7) | 0.41 | Within (increase of shooting performance in nfb feedback condition) |
| 9 | Wang and Hsieh, 2013 | Theta+ | 27.30(2,56) | 0.49 | Interaction (Phase (pre/post training) X flanker type. Significant only in nfb condition) |
| 9 | Wang and Hsieh, 2013 | Theta+ | 0.05(2,56) | 0.002 | Interaction (not-significant) (Phase (pre/post training) X flanker type in control condition) |
| 9 | Wang and Hsieh, 2013 | Theta+ | 10.03 (1,28) | 0.26 | Interaction (phase X cue only in ONFT condition) |
| 9 | Wang and Hsieh, 2013 | Theta+ | 66.69 (1,28) | 07 | Interaction (phase X conflict in ONFT and YNFT conditions) |
| 9 | Wang and Hsieh, 2013 | Theta+ | 10.32 (1,28) | 0.27 | Main (change in Stenberg recognition task pre to post nfb training in ONFT condition) |
